# Health care professionals’ perceptions of factors influencing the process of identifying patients for serious illness conversations: A qualitative study

**DOI:** 10.1177/02692163221102266

**Published:** 2022-06-21

**Authors:** Sofia Morberg Jämterud, Anna Sandgren

**Affiliations:** 1 Department of Thematic Studies, Linköping University, Linköping, Sweden; 2 Center for Collaborative Palliative Care, Faculty of Health and Life Sciences, Linnaeus University, Växjö, Sweden

**Keywords:** Communication, healthcare professionals, palliative care, qualitative research, serious illness conversations

## Abstract

**Background:**

The Serious Illness Care Programme enables patients to receive care that is in accordance with their priorities. However, despite clarity about palliative care needs, many barriers to and difficulties in identifying patients for serious illness conversations remain.

**Aim:**

To explore healthcare professionals’ perceptions about factors influencing the process of identifying patients for serious illness conversations.

**Design:**

Qualitative design. A thematic analysis of observations and semi-structured interviews was used.

**Setting/participants:**

Twelve observations at team meetings in which physicians and nurses discussed the process of identifying the patients for serious illness conversations were conducted at eight different clinics in two hospitals. Semi-structured interviews were conducted with three physicians and two nurses from five clinics.

**Results:**

Identifying the right patient and doing so at the right time were key to identifying patients for serious illness conversations. The continuity of relations and continuity over time could facilitate the identification process, while attitudes towards death and its relation to hope could hinder the process.

**Conclusions:**

The process of identifying patients for serious illness conversations is complex and may not be captured only by generic tools such as the surprise question. It is crucial to address existential and ethical obstacles that can hinder the identification of patients for serious illness conversations.

**What is already known about the topic?**

- Serious illness conversations promote patients’ possibility of receiving care that is in accordance with their wishes and priorities.
- Identifying patients for serious illness conversations remains difficult even when palliative care needs are identified.

**What this study adds?**

- Identification of patients for serious illness conversations is a process influenced by a multitude of factors, such as the patients’ palliative care needs, continuity in patient–professional relations and continuity of staff.
- Highlights the hesitation of non-palliative care professionals in identifying the patients for serious illness conversations due to existential and ethical concerns, such as fear of taking away hope.

**Implications for practice, theory or policy.**

- Identifying patients for serious illness conversations is a complex process involving several factors and is not limited to using generic tools, such as the surprise question.
- Identifying the right patient at the right time involves existential and ethical concerns which may impact healthcare professionals’ willingness to identify patients and offer serious illness conversations.
- Further research is needed on how health care professionals’ values and attitudes influence the identification process.

## Introduction

The Serious Illness Care Programme is a model which includes serious illness conversations for patients and family members with the goal that every seriously ill patient will have better and earlier conversations with their clinicians about their goals, wishes and priorities that will inform their future care.^1^ During recent years, a growing body of research on the Serious Illness Care Programme has shown that, when carried out well, these serious illness conversations promote shared decision making and the possibility for patients to receive care that is in accordance with their wishes and priorities.^1–3^ Furthermore, there is a connection between serious illness conversations and anxiety reduction.^4^ Many patients living with serious illnesses are open to talking about care options, values and goals in the end of life, and they find such conversations valuable.^5,6^ However, communication between physicians and patients about the patients’ care preferences in the end of life often does not happen^7^ or happens very late in the course of illness.^8^ This negatively impacts patients’ care in terms of enabling patients’ own goals and wishes.^9^ Serious Illness Care Programme has attained good results in qualitative improvement, such as improving the comfort of clinicians in holding serious illness conversations.^10,11^ The ‘serious illness conversation guide’ is a central tool in the programme,^12,13^ and has been useful in capturing vital information provided by the patient^14^ and providing the clinician with a concrete tool in holding such conversations.^15,16^ Non-palliative care clinicians have also enhanced their skills in conducting conversations, if educated and coached.^17–19^ To identify when serious illness conversations are of benefit to patients is crucial yet difficult.^20,21^ Even when palliative care needs are identified, barriers to carrying out serious illness conversations remain.^22^ The World Health Organization (WHO) defines palliative care as an approach that focuses on improving the quality of care for seriously ill patients as well as for their family members.^23^ To identify patients, the surprise question – ‘Would you be surprised if the patient died within 12 months?’ – has been used as a screening tool within the Serious Illness Care Programme.^10,21,24,25^ However, research has called for methods that can identify patients who are in need of serious illness conversations due to, for example, poor quality of life and not only methods based on prediction of mortality.^21^ More research is needed to understand the factors that can impact the identification of patients for serious illness conversations. Therefore, the aim of this study was to explore healthcare professionals’ perceptions about factors influencing the identification process of patients for serious illness conversations.

## Methods

### Study design

This study had a qualitative study design, and data were collected through a combination of observations (December 2018–April 2019) and semi-structured individual interviews (September 2019–January 2020). The benefit of observation studies in healthcare settings has been established as it enables, for example, the study of social processes.^26,27^ The study was guided by the Standard for reporting qualitative research (SRQR).^28^

### Setting

During 2018–2019, an adapted version of the Serious Illness Care Programme^12^ focussing on specialist physicians was implemented as a new work method at two acute care hospitals serving almost 200,000 habitants in a region in the south of Sweden. During the implementation of the Serious Illness Care Programme, several clinics arranged specific team meetings with physicians and nurses to discuss the implementation and process of identifying patients for serious illness conversations in their clinics. Some clinics chose to offer more than one team meeting in order to facilitate attendance for their health care professionals. The ‘surprise question’^24^ was promoted as a tool for identifying which patients could benefit from the conversations. The discussions during the team meetings covered both how identification could be done of the specific patient groups on a general level and examples of specific patients to facilitate the identification of these patients groups.

### Population

A broad range of clinics participated in the implementation of the Serious Illness Care Programme. However, it was the clinics themselves who decided whether they were going to organise team meetings as part of the implementation process. The inclusion criteria for the study was clinics which organised these team meetings. Inclusion criteria for the interviews was physicians and nurses who had attended the observed team meetings. Exclusion criteria were clinics that were not involved in the implementation or clinics that were involved but had decided not to organise team meetings.

### Sampling

A variety of clinics were selected in order to gain insights from different perspectives on the implementation and on the question on patient identification. Interviewees were collected through purposeful sampling^29^ from the participants in the team meetings in order to include different professions and different clinics.

### Data collection

Data were collected through a combination of observations and semi-structured individual qualitative interviews. Observations were conducted by the first author. The researcher was not involved in the discussions in the team meetings; she only focussed on listening and watching. An observation guide was used (Supplemental Material) to structure the fieldnotes. The guide covered ‘What criteria are considered grounds for identifying which of the patients should be offered a serious illness conversation?’, ‘Ethical and existential concerns in relation to identification’ and ‘What facilitates or hinders identification?’ The researcher also took fieldnotes of what happened and what other topics were discussed. Detailed fieldnotes were written and transcribed into 17 pages of text. The team meetings lasted between 45 min and 2 h; the total duration of observations was 14 h.

An initial analysis of the data from the observations was carried out by the first author and then discussed with the second author. In order to gain more detailed understanding of the identification process and the first preliminary analysis of the observations, we decided to complement the data with individual interviews. A semi-structured interview guide was used (Supplemental Material), which included the same topics as those covered in the observation guide. However, the design of the interview guide was also informed by the first preliminary analysis of the observations. The same interview guide was used in all interviews. Follow-up questions were posed to gain a deeper understanding.^30^ The interviews were conducted in a hospital setting, by the first author who is highly experienced in qualitative research. The interviews lasted between 17 and 40 min, were recorded, transcribed verbatim and pseudonymized.

### Data analysis

Thematic analysis^31^ was conducted on all data, including the transcribed field notes and transcribed interviews. The transcriptions were read independently of each other; then the first author carried out a detailed coding of the data and identified sub-themes based on this coding. Sub-themes were then clustered into broader patterns of meaning, that is, themes. The authors discussed the analysis and the themes regularly. The aim of this study steered the thematic analysis whilst also remaining open to other findings. The interview data did not bring out new themes but complemented and deepened the analysis from the observations. For this reason, the number of interviewees was deemed sufficient and no more interviews were conducted.

Regarding positionality of the researchers, the second author was responsible for the research on the implementation of the Serious Illness Care Programme while the first author, who conducted the data collection in this study, was part of the research group. However, none of the researchers was involved in the direct implementation of the programme at the hospitals.

### Ethical issues and approval

The Swedish Ethical Review Authority approved the study (Dnr 2018/540-31). Participants provided written informed consent prior to the interviews. They were informed that they could withdraw at any time, confidentiality would be ensured and about participation being voluntary. Permission for observations was granted by the hospital director, and written information was sent to the heads of all clinics. Personal data confidentiality was obtained, and no patient-related information was collected.

## Results

In total, 12 different team meetings were observed at eight different clinics (medicine *n* = 2, paediatric *n* = 1, surgery *n* = 5). Three team meetings were observed at medicine clinics (18 physicians; 12 nurses); two team meetings at a paediatric clinic (11 physicians; 1 nurse); seven team meetings at surgery clinics (61 physicians; 9 nurses). Totally 90 physicians and 22 nurses attended the team meetings. The groups ranged in size from 4 to 25 professionals. The majority of the physicians had previously participated in a 1-day communication training course together with actors, focussing on communication skills and how to use the serious illness conversation guide. The role of the nurses could be involvement in the identification of the patients and they could invite the patients for conversations with the physicians. After the observations were conducted, individual interviews were done with three physicians (two women and one man) and two nurses (two women).

**Figure 1.**
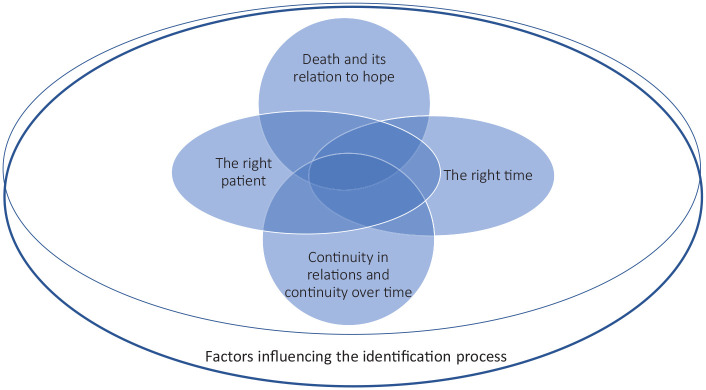
The complexity of the identification process of patients for serious illness conversation.

The identification process of patients for serious illness conversations was influenced by:

*the right patient, the right time, continuity in relations and continuity over time, and death and its relation to hope.* Although presented separately, they are interrelated themes (Figure 1).

### The right patient

Healthcare professionals’ perceptions about which patients would benefit from serious illness conversations are important for identification. Physical aspects as well as social and psychological aspects are woven into decisions. These views on what characterises ‘the right patient’ for identification should be regarded against the background that the study covers a broad spectrum of clinics (no specialist palliative care clinics).

However, the main characteristic of the right patient is physical deterioration, and the identification of patients for serious illness conversations can go hand in hand with identification of palliative care needs. The descriptions cover aspects such as ‘the patient is in a palliative phase’, ‘patients who regularly come to the ward and where their gradual deterioration is noticeable’, ‘treatment doesn’t help’ and ‘the patient neither improves nor deteriorates’. The patients themselves can also signal to healthcare professionals that they are experiencing severe physical deterioration, and that they do not want more treatment since they suffer because of it.

Another characterisation of the right patient concerns social aspects, such as changes of social behaviour: ‘You see a pattern. And not just in their illnesses but in their social lives and in their behaviours, in their attitudes’. This can regard, for example, that the patient: ‘has become discouraged and more introverted as an individual. This is true for most patients . . . they do not want to talk as much as they have before’. Additionally, the characterisation regarding psychological aspects is taken into consideration, such as that: ‘the patient is more worried and contacts the health services more frequently, is absent-minded and undergoes personality changes’.

At clinics where patient groups can be regarded as severely ill, but not palliative, offering serious illness conversations is more challenging. It can be more difficult to determine why they should offer the conversations, especially since the surprise question of 1 year survival is not applicable for identification of such a group of patients:

I: One could use the surprise question [. . .] Did that cause strain?

P: ‘You know, it is difficult to say. When were they in that stage? It just felt weird’ (P1).

The surprise question didn’t seem to be a suitable one for identification of all the patient groups.

### The right time

The previous theme of the right patient ties to the second theme – the right time. This refers to where in the patient’s illness trajectory the conversations would be of value to the patient, with the main reason being that the patient would not survive treatment:

Perhaps a year prior to the patient dying, even though you cannot know when they are going to die. But you come in early, but at the same time so late that you understand that the patient will not survive this (P3).

Specifically, the right time is considered highly important in relation to identification. If the conversation is offered too early, there is a risk of the patient losing hope: ‘One must be very careful and not offer serious illness conversations too early in the process since one then takes away hope’. Furthermore, the risk of offering conversations too early can lead to the patients not benefitting from the conversations. Healthcare professionals may consider it appropriate to offer a conversation based on their knowledge of the patients’ condition, but the patients might not consider themselves seriously ill:

However, at some point, you need to say stop, and now we thought we had come to a point where she [the patient] did not respond well to her own treatments. Unfortunately, this is not something she realises on her own, and I think that is why she assumed that it [the serious illness conversation] was a way to stop [the treatment] when actually. . .we wanted to clarify whether we share the same view on the situation (P2).

These clashes in views about the seriousness of an illness and the appropriateness of offering conversation ties into the view that certain conditions are, to a greater extent, understood as connected to death. Certain diagnoses are described as providing ‘natural inputs’ to serious illness conversations. The clearest example of this is cancer. Other illnesses, such as heart failure, are not similarly perceived. Therefore, it can be more difficult for some patients, who do not understand the severity of their disease, to realise why they are offered a serious illness conversation at that specific time.

### Temporality connecting ‘the right patient’ and ‘the right time’

Temporality can be regarded as an important aspect in the two above described themes. It has already been pointed out that physical, social and psychological aspects are woven into the decision when it comes to determining which patients would benefit from serious illness conversations. This can include temporal aspects since certain characteristic of *the right patient* such as ‘changes of social behaviour’ or ‘physical deterioration’ requires observation over time. However, in the descriptions of identification of patients for serious illness conversations a great deal is influenced by *when* it would be beneficial for the patient to be offered a conversation. Especially in the second theme ‘the right time’ there is a focus on these descriptions which includes *timing*.

### Continuity in relations and continuity over time

The third theme concerns continuity in relations and continuity over time as essential preconditions for identifying the patients for serious illness conversations.

It is important with established relationships and continuity in the relations between the patients and the healthcare professionals since the conversations can be difficult and challenging for both parties: ‘But then, I’m also thinking that it’s important not to barge in. These kinds of conversations somehow require a relation’. Offering conversations to a patient where there is no established relation is seen unsuitable because one touches upon sensitive topics. Continuity over time is also important in identifying patients since healthcare professionals, as a team, can follow the patients’ process of deterioration.

Identification is also facilitated by a continuity of staff over time: ‘I believe continuity of the staff is important. Without that, it [identification of patients] would be rather tricky’. However, when healthcare is fragmented and healthcare professionals meet patients sporadically, it is more difficult and sometimes ‘impossible’ to identify which patients would benefit from serious illness conversations.

### Death and its relation to hope

Death and its relation to hope entails ethical concerns in relation to identifying patients for serious illness conversations. The general line of thought is that hope is of significance for survival, and if offering serious illness conversations the topic of death and dying can be brought up, which is regarded as possibly having a negative impact on patients’ hope. Hope is often connected to survival, and if healthcare professionals acknowledge and verbalise concerns about death and dying, there is a fear of ‘awakening’ the patients’ or relatives’ thoughts of time being limited, taking away hope and, consequently, upsetting them. This is considered to be of great seriousness: ‘Since we know that hope is of importance in relation to survival, you don’t want to snuff out the patient’s hope’. However, some also claim that hope can transform in character and does not necessarily mean survival, and that approaching the topic of death does not necessarily mean taking away hope:

If you have already here [at the hospital] talked about this potentially not turning out the way you thought, you might not get through this. They have both heard it. The patient does not think he will survive or the wife does not think that he will survive. So, I think you are doing something good. . . Then they can concentrate more on having a good time together, this remaining time. So, I do not think that one is snuffing out someone’s hope (P3).

The idea that serious illness conversations take away hope is also said to relate to healthcare professionals’ own resistance and fear of talking about death and dying. Additionally, death is often regarded as ‘the greatest fear’ and ‘a failure’ among healthcare professionals.

## Discussion

### Main findings

We found that the identification process of patients for serious illness conversations is influenced by identifying the right patient as well as identifying the patient at the right time. The continuity of relations and continuity over time could facilitate the identification process, while attitudes about death and its relation to hope could hinder the process.

### Strengths and limitations

A strength of our study is that we combined data from both observations at team meetings and individual semi-structured interviews. This allowed us to capture the healthcare professionals’ spontaneous discussions on the identification process in the team meetings and then obtain a more in-depth understanding about the process in the individual interviews due to the semi-structured design. The relatively small sample size in the interviews could be seen as a limitation. However, these were done as a complement to the observations. This study is based on the implementation of the Serious Illness Care Programme in two acute care hospitals, making generalisability challenging to other contexts. Although our results show several aspects which influence the identification process, there may be other possible factors that facilitate or hinder the identification process in other contexts.

### What this study adds

Our results demonstrate that for certain patient populations, instead of the criteria of 1-year survival, clinicians use information about patients’ medical, social and/or psychological symptoms to identify patients for serious illness conversations. This is an important result in relation to previous research which called for methodologies to make it possible to identify a broader range of patients in need of palliative care than trying to predict the life span for a seriously ill patient^13^ by using the surprise question.

Furthermore, other identification strategies than the surprise question are necessary in identifying patients in early palliative stages.^10^ In our results, the focus on palliative care needs in the identification process is central. Based on this, it can be suggested that serious illness conversations can be seen as a form of palliative care communication and a way to implement palliative care at an earlier stage in patients’ disease trajectory.^32^ This is significant since our study is based on a non-specialist palliative care context.

The process of identifying the right patient at the right time involves both ethical and existential aspects. The concern about taking away patients’ hope relates to identifying patients at ‘the right time’. To decide to identify patients for serious illness conversations is connected to whether healthcare professionals see the benefit of offering these as well as a hesitation in offering these due to worry for harming patients. One example shown in the result is the view that serious illness conversations can entail the topic of death and dying, and in addressing this topic, one risks taking away the patient’s hope. Our results are in line with previous research describing professionals’ fear of taking away patients’ hopes.^33,34^ Our results could also be set in a broader context. The focus in healthcare is often on cure, a position that can be ‘welcomed’ by patients because focussing on what can be treated is often less burdensome than the existential suffering caused by facing death and dying.^35^ However, studies have shown that patients often want to talk about these issues,^36^ and if clinicians are trained, these conversations do not decrease the patients’ hope.^37^ Research has also shown that the patient’s experience of hope is not limited to possibilities for treatment but that other factors, such as knowing that one will receive good care and be able to make one’s own decisions, are of importance.^38^

Our results highlight the necessity of addressing existential obstacles that can hinder the identification process. This may be particularly important when introducing this programme to non-specialist palliative care contexts. Palliative care is based on a holistic view of the human being, in which care for existential concerns, such as death, are integral parts of the meaning of good care and addressing existential needs is an area of responsibility of care.^39^ However, this may not be the case for many clinics that can find this challenging and where addressing existential concerns might not be regarded as an area of responsibility of care. This may have implications for identification. When healthcare professionals make themselves available for sharing the patients’ existential experiences, this can become an existential challenge for them.^35^ Future research could focus on examining how these existential aspects could influence health care professionals’ willingness to invite such conversations, and hence, on the willingness to identify patients for such conversations.

Research on serious illness conversation has pointed to the importance of training in areas such as: conducting conversations, communication skills, sharing prognosis and responding to emotions.^10,16^ Such training improves the competence of physicians in conducting these conversations.^16^ Physicians who have received training are also more likely to conduct conversations without harming the patients.^22^ Our study highlights that existential and ethical concerns can arise in relation to inviting as well as holding serious illness conversations. These areas could be of importance to address in training since they can have implications for the identification of patients for serious illness conversations. Approaching questions about hope and death is not only about conversational methodology but also about profound human values, addressing these questions is part of the area of responsibility of care.^39^

Further research is needed on the factors that influence the identification process as well as the complexity of the identification process such as to what extent the patients benefit from there being continuity between patient and physician, in order to benefit from such conversations. Previous research has also highlighted that more studies are needed on how providers of serious illness conversations are impacted.^22^ We agree with this conclusion, and our results highlight the need for further research on the connection between the impact on providers regarding existential and ethical concerns and their willingness to identify patients for serious illness conversations. We also suggest that further research on existential and ethical challenges in relation to serious illness conversations is needed in clinical practice to successfully and sustainably implement this model.

## Conclusions

The identification process of patients for serious illness conversations is influenced by identifying the right patient as well as identifying the patient at the right time. The continuity of relations and continuity over time could facilitate the identification process, whereas attitudes towards death and its relation to hope could hinder the process. The identification process is more complex compared to using generic identification tools and addresses a broad range of concerns, such as continuity in patient-professional relationships and the patient’s palliative care needs. This study highlights the necessity to also address and research existential and ethical obstacles, such as the fear of taking away hope, which can hinder the identification of patients for serious illness conversations.

## Supplemental Material

sj-pdf-1-pmj-10.1177_02692163221102266 – Supplemental material for Health care professionals’ perceptions of factors influencing the process of identifying patients for serious illness conversations: A qualitative study Click here for additional data file. Supplemental material, sj-pdf-1-pmj-10.1177_02692163221102266 for Health care professionals’ perceptions of factors influencing the process of identifying patients for serious illness conversations: A qualitative study by Sofia Morberg Jämterud and Anna Sandgren in Palliative Medicine

sj-pdf-2-pmj-10.1177_02692163221102266 – Supplemental material for Health care professionals’ perceptions of factors influencing the process of identifying patients for serious illness conversations: A qualitative study Click here for additional data file. Supplemental material, sj-pdf-2-pmj-10.1177_02692163221102266 for Health care professionals’ perceptions of factors influencing the process of identifying patients for serious illness conversations: A qualitative study by Sofia Morberg Jämterud and Anna Sandgren in Palliative Medicine
