# Supplementary material for: Health care professionals’ perceptions of factors influencing the process of identifying patients for serious illness conversations: A qualitative study
Source: Palliat Med. 2022 Jun 21;36(7):1072–9. doi: 10.1177/02692163221102266 (PMC9247430; doi:10.1177/02692163221102266)
Supplement: sj-pdf-1-pmj-10.1177_02692163221102266 – Supplemental material for Health care professionals’ perceptions of factors influencing the process of identifying patients for serious illness conversations: A qualitative study [file sj-pdf-1-pmj-10.1177_02692163221102266.pdf]

SUPPLEMENTARY FILE.
SEMI-STRUCTURED INTERVIEW-GUIDE

Title of article: "Health care professionals' perceptions of factors influencing the process of identifying patients for serious illness conversations: A qualitative study"

Due to the semi-structured format of the interviews the topics and *examples* of questions are stated.

General questions

1. Could you describe your understanding of serious illness conversations?

Identification of patients

2. Which groups of patients do your regard could benefit from serious illness conversations?
3. Are there groups of patients you do not regard should be identified for serious illness conversations?
4. How do you identify patients for serious illness conversations?
5. Did you make use of the surprise question in identification and was that a useful tool?
6. Could you please describe what you think facilitates the identification of a patient for serious illness conversations?
7. Could you please describe what you think hinders the identification of a patient for serious illness conversations?

Ethical and existential aspects of identifying patients for serious illness conversations

8. What would you describe as the pros and cons for the patient in being identified for and offered a serious illness conversation?
9. Did you see any ethical problems with offering patients a serious illness conversation?

Work methods and experiences

10. Could you please describe how your team has been working with the Serious Illness Program?
11. Could you please describe how you yourself experience the work with serious illness conversations?

Other questions

12. Is there anything we haven't discussed that you would like to add?
