# Supplementary material for: Health care professionals’ perceptions of factors influencing the process of identifying patients for serious illness conversations: A qualitative study
Source: Palliat Med. 2022 Jun 21;36(7):1072–9. doi: 10.1177/02692163221102266 (PMC9247430; doi:10.1177/02692163221102266)
Supplement: sj-pdf-2-pmj-10.1177_02692163221102266 – Supplemental material for Health care professionals’ perceptions of factors influencing the process of identifying patients for serious illness conversations: A qualitative study [file sj-pdf-2-pmj-10.1177_02692163221102266.pdf]

SUPPLEMENTARY FILE. OBSERVATION GUIDE

Title of article: "Health care professionals' perceptions of factors influencing the process of identifying patients for serious illness conversations: A qualitative study"

Date:
Time:
Location:
Length of the meeting:
Name of researcher:
Clinician leading the meeting:
Number of participants and their professions:

1. What criteria are considered grounds for identifying which of the patients should be offered a serious illness conversation?

2. Subjects regarding ethical and existential concerns in relation to identification.

3. What facilitates or hinders identification?

4. The observers' own reflections of the process
